# Ten year outcomes of participants in the REACT (Randomised Evaluation of Assertive Community Treatment in North London) study

**DOI:** 10.1186/s12888-014-0296-6

**Published:** 2014-10-24

**Authors:** Helen Killaspy, Laia Mas-Expósito, Louise Marston, Michael King

**Affiliations:** Division of Psychiatry, University College London (UCL), Charles Bell House, 67-73 Riding House Street, London, W1W 7EJ UK; Research Associate, Research Unit, Centre d’Higiene Mental Les Corts, c/Numància 103-105 Baixos, 08029 Barcelona, Spain; Research Department of Primary Care and Population Health, UCL Medical School, London, NW3 2PF UK; UCL PRIMENT Clinical Trials Unit, UCL Medical School, Upper 3rd Floor, Royal Free Campus, Rowland Hill Street, London, NW3 2PF UK

**Keywords:** Assertive Community Treatment, Long term outcomes

## Abstract

**Background:**

A previous randomised controlled trial that investigated Assertive Community Treatment (ACT) in the UK (the REACT Study) found no clinical advantage over usual care delivered by Community Mental Health Teams (CMHTs) at 18 and 36 month follow-ups. No studies have investigated long term clinical and social outcomes for patients receiving ACT.

**Method:**

We investigated inpatient service use, social outcomes, service contact and adverse events for the 251 REACT study participants 10 years after randomisation through case note review. Data were analysed using regression models adjusted for original treatment group allocation and changes in treatment group.

**Results:**

We found no statistically significant differences in outcomes by original treatment group over the 10 years. Those whose care remained with ACT, or transferred to ACT or forensic services, had more inpatient days over the 10 years (coefficient 223, 95% CI 83 to 363, p = 0.002) than those whose care remained with the CMHTs or were discharged to primary care. Being subject to a Community Treatment Order was associated with a greater chance of being under ACT at 10 year follow-up (OR 6.39, 95% CI 2.98 to 13.70, p <0.001).

**Conclusions:**

The ACT teams in this study showed no clinical advantage over usual care provided by CMHTs at 10 year follow-up. We also found that the ACT teams accrued patients from the original study sample who had more complex needs than those who remained with or transferred to the CMHTs or primary care during this period. Further well conducted trials are needed to identify the most cost-effective approaches to supporting successful community living and optimum long term outcomes for this group.

## Background

Assertive Community Treatment (ACT) teams were implemented across England from 1999 onwards as part of the National Service Framework for Mental Health [1]. These teams provide flexible and intensive, home based support to people with severe mental health problems who are high users of inpatient care and have problems engaging with standard mental health services [2]. There is good evidence for their clinical efficacy when compared against standard outpatient care in terms of reducing the need for inpatient care and associated costs [3]. However, these advantages have not been replicated where “standard” care delivers a more intensive form of case management that replicates some of the key components of ACT [3-5]. However, meta-analysis of trials of intensive case management have concluded that the clinical benefits (particularly reduced inpatient service use) are most evident where there is higher fidelity to the ACT model and higher use of inpatient care in the local population [3,4]. The Randomised Evaluation of Assertive Community Treatment in North London (REACT) study assessed outcomes for 251 people randomly assigned to receive either ACT or standard case management from community mental health teams (CMHTs). Eighteen months after randomisation, no clinical advantage for ACT was found in terms of inpatient service use, but ACT patients were more satisfied and better engaged with services [5]. A further follow-up 18 months after the trial ended (at 36 months) replicated these main results, though by then 20 of the original ACT and 20 original CMHT participants had changed study arms [6]. Selection criteria for participants in the REACT study included high use of inpatient care and the comparison CMHTs had high fidelity for only one of the key ACT components (offering a time unlimited service).

Given that we had previously found no difference in inpatient service use between participants receiving ACT and comparison CMHT services at 18 and 36 month follow-up, the wisdom of further follow-up could be questioned. However, no studies of ACT have investigated outcomes beyond 36 months and it is not known whether receipt of this more intensive support might lay foundations for a better life course and less need for inpatient services, with benefits being manifest some years later. Furthermore, the most recent Cochrane review of intensive case management advised that studies of ACT should also assess specific social outcomes (such as employment) rather than assessing social function using standardised measures [3].

The aim of this study was to investigate whether ACT was associated with better long term clinical and social outcomes and the predictors of ongoing need for ACT. We therefore investigated inpatient service use and social outcomes for participants in the REACT study 10 years after randomisation. We expected that many participants would have changed from their original treatment allocation by this point and that this would need to be accounted for in our analysis. Although the teams did not use any standardised criteria for transfers, in order for an ACT patient to be accepted by a CMHT (that provides less intensive support than the ACT teams) or discharged to primary care, we felt it reasonable to assume that they would have stabilised (be better engaged and causing less concern to the ACT team). Similarly, patients who transferred from a CMHT to the ACT team were likely to be those where there were ongoing difficulties in engagement, concerns about risk and recurrent admissions to hospital. We were also aware that some patients would have been transferred to forensic services due to the severity of these issues. A transfer of care variable reflecting this was therefore included in our statistical models. We also investigated predictors of remaining in ACT or being transferred to forensic services at 10 year follow-up in order to identify factors associated with ongoing use of these more intensive services.

The original REACT study and the further follow-up studies were approved by the Camden & Islington Community and Royal Free Hospital Research Ethics Committee (Ref. 99/93).

## Method

The REACT study was carried out with full adherence to CONSORT guidelines for the management of randomised controlled trials. The methods and results 18 months after randomisation have been reported elsewhere [7]. In brief, the 251 participants were recruited from all CMHTs in the London boroughs of Camden and Islington between July 1999 and July 2002. They were high users of inpatient care (at least 100 consecutive inpatient days or at least five admissions within the past two years; or at least 50 consecutive inpatient days or at least three admissions within the past year) who were living independently and whom the CMHTs had found problematic to engage over at least the previous 12 months. There were no differences between the two groups in clinical or social functioning at baseline.

Since including only consenting participants would render the results irrelevant to the service users most likely to be referred for ACT, the Research Ethics Committee approved randomisation and collection of case note and key informant data on all participants, whether or not they agreed to participate in the research interviews. Participants were randomly allocated on an equal basis to the care of one of the two local ACT teams or to continue with their CMHT. The fidelity of the ACT teams was independently assessed using the Dartmouth Assertive Community Treatment Scale [8] during the REACT study and found to be high for one team, “ACT like” for the other [7] and low for the CMHTs [9].

LME (co-author) collected data on participants’ contact with services, inpatient service use, use of the Mental Health Act and adverse events (deaths, incidents of self-harm, violence, imprisonment, homelessness and loss of contact with services) over the 10 years since randomisation. Details of employment/other occupation (supported employment/courses) and use of supported accommodation over the 10 years were also gathered as well as their current involvement in social/leisure activities and contact with family. Data were collected from paper and electronic case notes (implemented across the local mental health services in 2006) except for three participants who had moved out of the area and four participants whose care had been transferred to forensic mental health services. These participants’ data were gathered from their care co-ordinators by email or telephone. For a further two participants, no follow-up data could be collected as both paper and electronic records were missing.

### Participant flows

**Figure 1:**
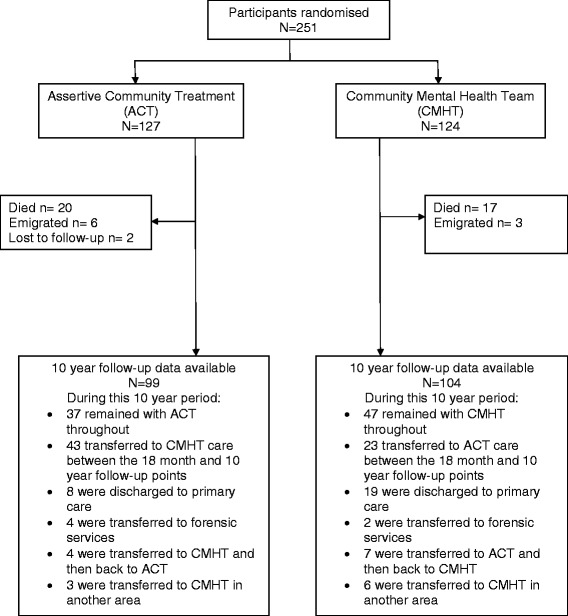
**REACT study participant flows 10 years after randomisation.**

The original REACT study required 250 participants to detect a difference of 60 (SD 169) bed days between the ACT and CMHT groups with 80% power. Of 251 study participants recruited, 127 were allocated to ACT and 124 to CMHT care. Eighteen months after randomisation, three ACT and four CMHT participants had died and one CMHT participant had emigrated, so primary outcome data for the original trial were available for 124 ACT and 119 CMHT participants. Ten years after randomisation, a further 17 ACT and 13 CMHT participants had died and 8 ACT and 2 CMHT participants had emigrated or Cere out of contact with mental health services. Hence 10 year outcome data were available for 99 ACT and 104 CMHT participants. Forty-three of the original ACT participants had been transferred back to the care of a CMHT, 4 had been transferred to forensic services and 8 had been discharged to primary care between the 18 month and 10 year follow-up. Of the CMHT participants, 23 had been transferred to an ACT team, 2 had been transferred to forensic services and 17 had been discharged to primary care during this period. See Figure 1 for further details.

### Data analysis

Our first analysis investigated whether participants in the REACT study who were randomised to receive ACT required less inpatient care over the 10 year follow-up period than those randomised to receive CMHT care. The total number of inpatient days used over the 10 years was compared using a generalised estimating equation (GEE) that was able to account for the fact that participants who died or emigrated during the 10 years would not have data available for collection at all follow-up time points. The model included participants’ total inpatient days used at 36 month and 10 year follow-up. Standard errors were created using bootstrapping with 5000 replications. Variables entered into the model included the original treatment allocation at recruitment into the trial (ACT or CMHT), inpatient days prior to recruitment into the trial (to control for those who were very high users of inpatient care [ACT median 441 (IQR 222, 834); CMHT median 445 (IQR 264, 773)], and a binary time varying variable that took into account changes in participants’ treatment between the end of the trial at 18 month follow-up and 10 year follow-up, as explained in the background (0 = those who had remained in the CMHT, transferred from ACT to CMHT or been discharged from ACT or CMHT to primary care; 1 = those who had remained in ACT, transferred to ACT from primary care or a CMHT or transferred to forensic services from an ACT team or CMHT).

We also investigated social outcomes at the 10 year follow-up point (employed or in supported employment or enrolled on an educational or vocational course; involved in social and/or leisure activities; having contact with family) using logistic regression. A variable for the treatment being received at 36 month follow-up (0 = CMHT or primary care, 1 = ACT or forensic) was included in the model along with a similar binary change of treatment variable (as described above) to account for subsequent transfers between services (time invariant, 36 months to 10 years). Transfers of care between 36 month and 10 year follow-up were included rather than between 18 month and 10 year follow-up because social outcome data were only collected at 36 month and ten year follow-up. Due to low numbers of people employed or engaged in social or leisure activities at 10 year follow-up, these three variables were combined.

Factors associated with being under the care of an ACT team or forensic service at 10 year follow-up were also investigated using logistic regression. Univariate analysis was used to identify variables for inclusion in these models.

## Results

Descriptive data on all 10 year outcomes are shown in Table 1 by original treatment allocation.

**Table 1:** **Ten year outcomes for REACT study participants by original treatment allocation**

**Outcome**	**Assertive community treatment**			**Community mental health team**		
	**N = 99**			**N = 104**		
**Inpatient service use**	**Mean (SD)**	**Median**	**IQR**	**Mean (SD)**	**Median**	**IQR**
Total inpatient days	1049 (1037)	671	301, 1515	948 (950)	592	256, 1333
Total admissions	6 (5)	5	2, 8	5 (4)	5	2, 7
Days per admission	236 (422)	119	70, 286	215 (304)	124	64, 239
Involuntary admissions	4 (4)	3	1, 6	4 (4)	4	2, 6
**Admissions**	**N**	**n (%)**		**N**	**n (%)**	
Any admission	99	90 (91)		104	93 (89)	
Number of admissions	99			104		
*1 admission*		14 (14)			7 (7)	
*2 admissions*		6 (6)			8 (8)	
*> 2 admissions*		70 (71)			78 (75)	
Involuntary	99	84 (85)		104	92 (88)	
Intensive Care Unit	99	24 (24)		104	28 (27)	
Low Secure Unit	99	2 (2)		104	0 (0)	
Medium Secure Unit	99	6 (6)		104	5 (5)	
High Secure Unit	99	1 (1)		104	0 (0)	
Supervised discharge*	93	23 (25)		99	21 (21)	
**Social outcomes**	**N**	**n (%)**		**N**	**n (%)**	
Currently in employment or other occupation	99	31 (31)		104	39 (38)	
Any employment or occupation 36 months to 10 years	99	61 (62)		104	59 (57)	
Currently involved in social or leisure activities	81	38 (47)		80	30 (38)	
Currently in contact with family	84	60 (71)		85	50 (59)	
Currently living in supported accommodation	99	39 (39)		104	52 (50)	
Any supported accommodation last 10 years	99	64 (65)		104	58 (56)	
**Adverse events**	**N**	**n (%)**		**N**	**n (%)**	
Lost to follow-up^a^	83	8 (10)		76	12 (16)	
Homelessness	99	20 (20)		104	23 (22)	
Physical assault on another person^b^	99	51 (52)		104	62 (60)^c^	
Physical assault on a stranger	99	26 (26)		104	22 (21)	
Sexual offence	99	11 (11)		104	20 (19)	
Arson	99	12 (12)		103	13 (13)	
Prison	99	19 (19)		104	29 (28)	
Deliberate self-harm (DSH)	99	23 (23)		104	30 (29)	
Recurrent DSH^d^	99	8 (8)		104	7 (7)	

* Supervised discharge = Community Treatment Order or Section 25 of the Mental Health Act (1983).

^a^Lost to follow-up defined as no face-to-face contacts between staff and clients in previous three months (this variable is not measured over the last 10 years).

^b^The victim could be known to the perpetrator or a stranger.

^c^The most serious assault was one incident of manslaughter.

^d^Recurrent deliberate self-harm defined as at least five episodes in two years.

Our first analysis found that the initial randomisation group (ACT or CMHT) was not statistically significantly associated with total inpatient bed days over the 10 year period (see Table 2). However, those whose care remained with or transferred to ACT or forensic services during the 10 years (change variable group 1) used more inpatient days on average than those whose care remained under the CMHT, transferred from ACT to CMHT or were discharged to primary care (change variable group 0). Having been an inpatient for more days prior to randomisation was also associated with a higher number of inpatient days over the 10 years.

**Table 2:** **Linear regression with generalised estimated equation of total inpatient days used over the 10 years after randomisation into REACT study**

	**Coefficient**	**Bootstrap SE**	**95% CI**	**p**
Randomised to ACT	−34.61	51.16	(−179.30, 110.09)	0.639
Stayed with or transferred to ACT	223.01	71.38	(83.10, 362.92)	0.002
Inpatient days prior to randomisation	0.19	0.072	(0.05, 0.33)	0.009

None of the social outcomes assessed at 10 year follow-up were associated with treatment group at 36 months or change in treatment group from 36 months to 10 years (see Table 3).

**Table 3:** **Logistic regression: social outcomes at 10 year follow-up for participants of the REACT study**

	**Odds ratio**	**95% CI**	**p**
**Employed/course/social/leisure**			
ACT at 36 months	1.05	(0.53, 2.08)	0.88
Stayed with or transferred to ACT (36 months to 10 years after randomisation)	0.62	(0.31, 1.25)	0.18
**Family contact**			
ACT at 36 months	1.65	(0.82, 3.32)	0.16
Stayed with or transferred to ACT (36 months to 10 years after randomisation)	1.10	(0.53, 2.26)	0.81

Two factors were found to be associated with being in ACT or forensic care at 10 years. Those randomised to ACT originally were more likely to remain in ACT or be in forensic care at 10 years than those allocated to CMHT care originally (OR 2.89, 95% CI 1.49 to 5.60, p = 0.002). Those who were on a Community Treatment Order (CTO) at 10 year follow-up were more likely to be under ACT or forensic care at this point than those who were not on a CTO (OR 6.39, 95% CI 2.98 to 13.70, p < 0.001).

## Discussion and conclusions

We found no significant difference in 10 year outcomes for participants of the REACT study. Our findings therefore concurred with our previous results at 18 months and 36 months. After the original study ended, the ACT teams continued to acquire patients from the original REACT study sample who were allocated to CMHT care, whose needs were complex (difficult to engage in treatment and requiring recurrent admissions due to the severity of their symptoms and risk profile) and to transfer or discharge those who had stabilised. This probably explains why inpatient bed use was higher for those who remained with/transferred to ACT during the 10 years compared to those who remained with/transferred to CMHT. This is further supported by our finding that one of the two factors associated with being under the ACT team at 10 years was being on a CTO. Alternatively, ACT teams are perhaps more likely to admit their patients, to use CTOs, and to refer them to forensic services than CMHTs. However, given that the threshold for admission in inner London is high and that the ACT teams do not hold gatekeeping responsibility for admissions to inpatient or forensic services, this seems unlikely (gatekeeping of admissions to general inpatient services is the responsibility of the local crisis resolution teams and admissions to forensic services are usually directed by the criminal justice system or agreed after assessment by a forensic psychiatrist). It is however possible that the finding that those randomised to ACT originally were more likely to remain in ACT or be in forensic care at 10 year follow-up could have been due to ACT staff being reluctant to discharge clients who they had managed to engage with.

This study has an important limitation that needs to be considered when interpreting the results. Data were gathered from case notes and are therefore dependent on the quality of recording of events by staff. However, our data on inpatient service use are likely to be accurate due to the considerable administrative infrastructure in the NHS that primarily focuses on recording admission and discharge data and the legal requirement for this for detained patients. Since a very high proportion of patients in this study were involuntarily admitted, it is reasonable to assume that these data are robust. Similarly, the need for clinicians to ensure accurate recording of risk incidents means that our data on adverse events is also likely to be reliable. However, the recording of information on social outcomes is not subject to such close scrutiny and it is therefore possible that these were not recorded as accurately in the case notes. We may therefore have underestimated social gains that patients made, though we have no reason to suspect that this information would have been recorded differently by ACT and CMHT staff. A further limitation is that the study was carried out in one area of North London and the results may therefore not generalise to other settings and service contexts [10].

Our findings should not be interpreted as conclusive evidence that ACT does or does not “work”, but they do highlight the difficult task facing ACT teams and the ongoing need for specialist services for people with severe, longer term mental health problems. In keeping with our original assumption, it appears that after the trial ended, the ACT teams continuously accrued people with complex psychosis; around one quarter of those originally allocated to CMHT care required transfer to ACT. In addition, around one third of those originally allocated to ACT did not stabilise adequately for transfer to CMHT or primary care. The ACT teams were also taking on new clients who were not participants in the original REACT study but who met the original criteria (high users of inpatient care, with recurrent disengagement from CMHT care). There is now considerable disinvestment in ACT teams in England; many are being disbanded or reconfigured into a more “dilute” version of the ACT model which adheres less strictly to ACT model fidelity and delivers fewer of the key components [11]. Hybrid approaches that combine features of ACT and standard case management are popular in some European countries with similar mental health systems to the UK [12] but no trials investigating their efficacy have been carried out to date. There is ongoing international debate about whether ACT should devolve back to a more generic case management model in the UK [13-15]. What is certainly clear is that investment in community teams that are adequately resourced to deliver evidence based treatment and support to those with the most complex severe mental health problems is required. Further well conducted trials are needed to identify the most clinically effective approaches that can support successful community living and optimum long term outcomes for this group, whether this is through traditional ACT or other models of care.
